# Supplementary material for: Functional genomics implicates ebony in the black pupae phenotype of tephritid fruit flies
Source: Commun Biol. 2025 Jan 15;8:60. doi: 10.1038/s42003-025-07489-y (PMC11736145; doi:10.1038/s42003-025-07489-y)
Supplement: Supplementary file 1 — Supplementary Information [file 42003_2025_7489_MOESM1_ESM.pdf]

Functional genomics implicates *ebony* in the black pupae phenotype of tephritid fruit flies

by

Paulo and Nguyen *et al.*

Supplementary Figures 1 – 9

Supplementary Tables 1 – 6

Supplementary References

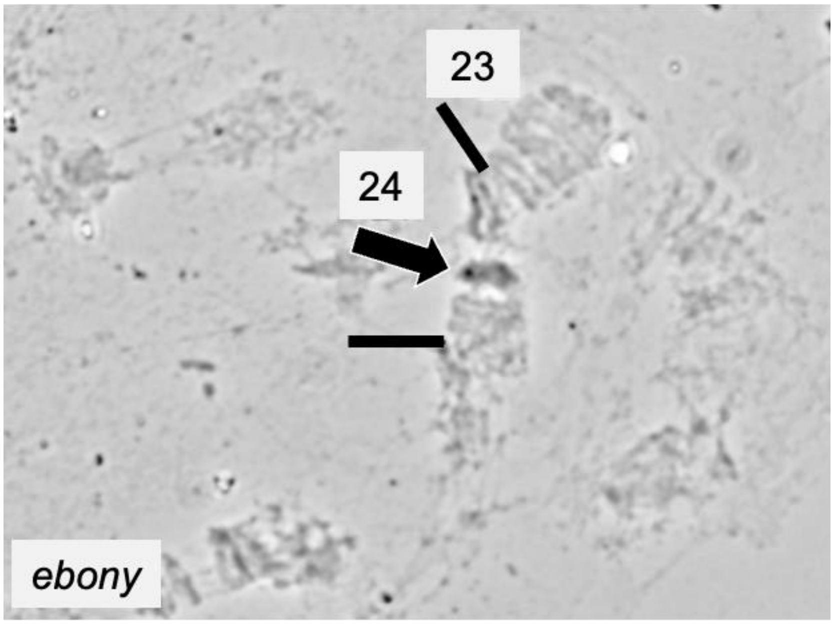

Supplementary Figure 1. *In situ* hybridization of *ebony* (arrowhead) in region 24 of the salivary polytene chromosome III (or mitotic chromosome 2) of *Anastrepha ludens*. Hybridizations were performed in duplicates and at least ten nuclei were analyzed per sample.

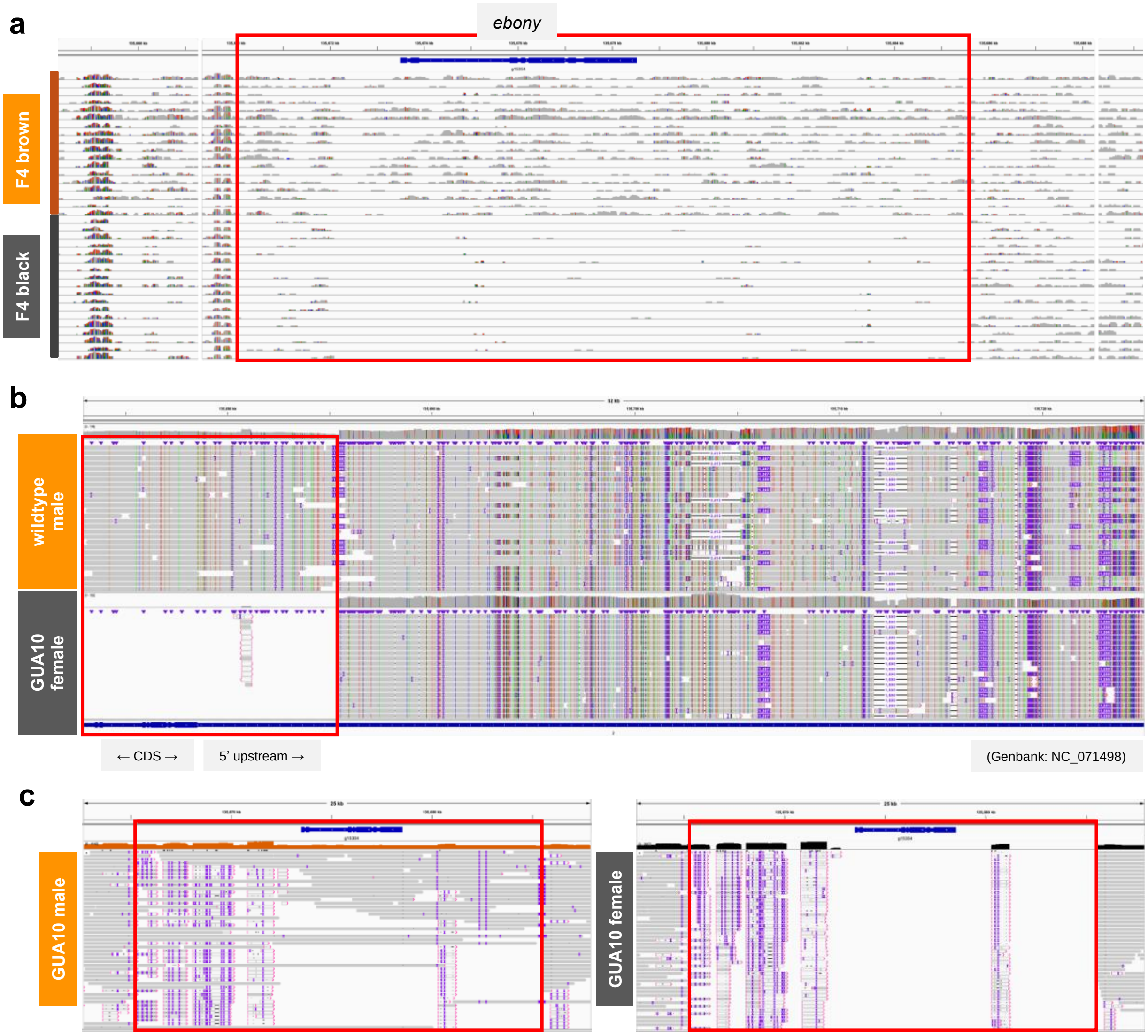

Supplementary Figure 2. Localization of the *bp*⁻ mutation within the *ebony* loci of the *A. ludens* GUA10 genetic sexing strain. **(a)** WGS short-read coverage comparison between brown ($bp^{+/+}$ or $bp^{+/-}$) and black ($bp^{-/-}$) pupae siblings from the F4 mapping population (n = 18 individuals per phenotype). **(b)** HiFi long-read mapping comparison between wildtype male ($bp^{+/+}$) and GUA10 female ($bp^{-/-}$). **(c)** HiFi long-read mapping comparison between GUA10 male ($bp^{+/-}$) and GUA10 female ($bp^{-/-}$). Differential read mapping between samples is highlighted within red boxes. Images are screenshots from the Integrative Genomics Viewer (IGV).

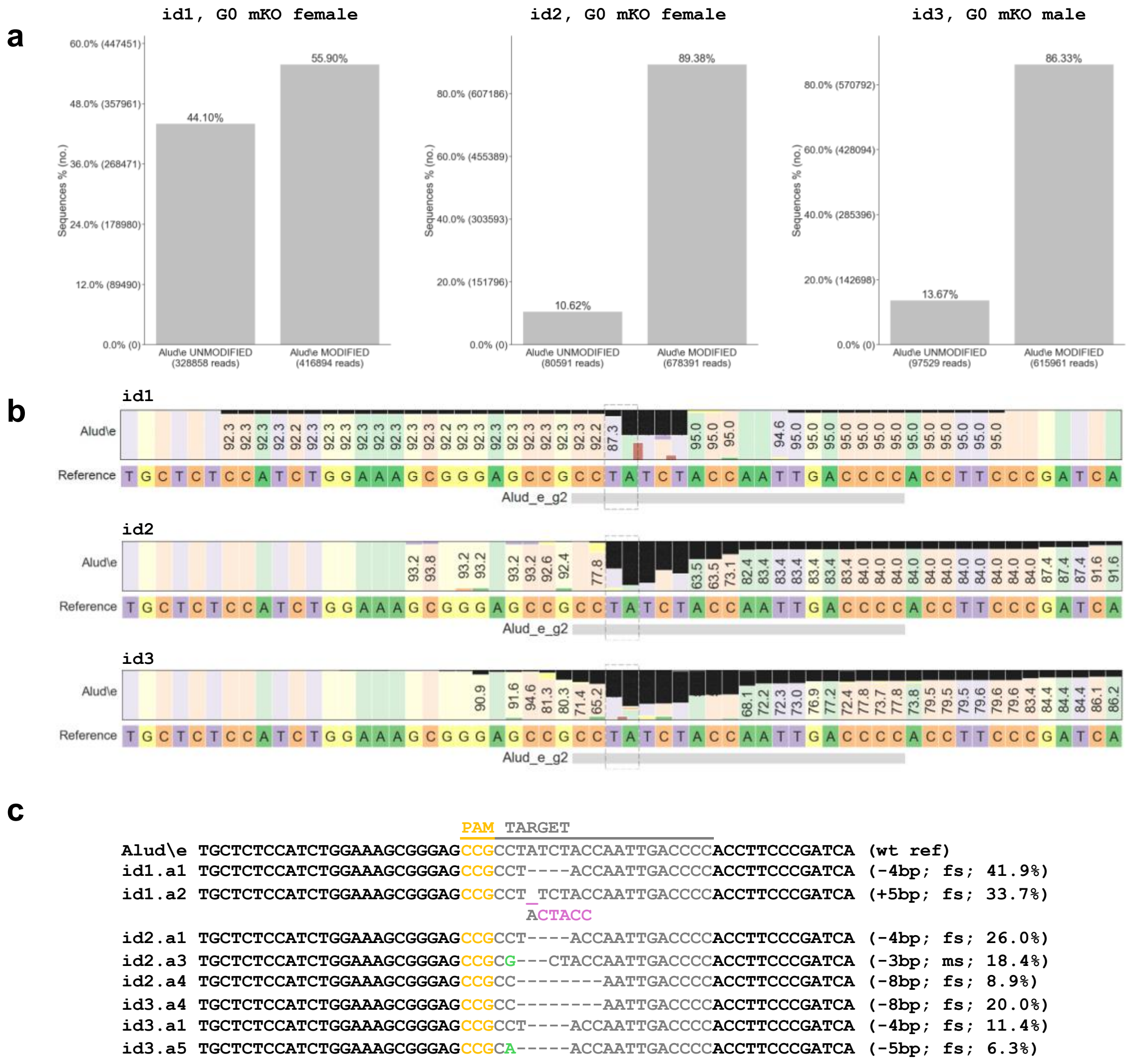

Supplementary Figure 3. Illumina genotyping of indexed amplicons surrounding Cas9 cut sites in three individuals (id1–3) of *A. ludens* G0 mosaic knockouts (mKO). The CRISPResso2 pipeline was used to assemble overlapping read pairs and detect indels. **(a)** Mutagenesis frequency as determined by the percentage of reads displaying modified alleles (Quantification window center = -3, Quantification window size = 2 bp). **(b)** Nucleotide distribution across the amplicon and percentage of each base relative to the wildtype reference amplicon (wt ref). Black and brown bars represent the percentage of deletions and insertions, respectively. **(c)** The most frequent allele variants found in mosaic flies. Percentages are relative to 713,490 – 758,982 mapped reads. Expected outcomes include a number of frameshift (fs) and missense (ms) mutations.

a

```
                                PAM  TARGET
Afra\e CTCAGTGTCCGAACTGTGGGGGCCGCTGATGTGTGGACTTTCTATTTTAGTTGTACCCAAAGTAATAACTA (wt ref)
        ..S..V..S..E..L..W..G..P..L..M..C..G..L..S..I..L..V..V..P..K..V..I..T..
G2.KO  CTCAGTGTCCGAACTGTGGGGGCCGCTG-------GACTTTCTATTTTAGTTGTACCCAAAGTAATAACTA (-7bp)
        ..S..V..S..E..L..W..G..P..L.-------.D..F..L..F..*..L..Y..P..K..*..*..L.
```

b

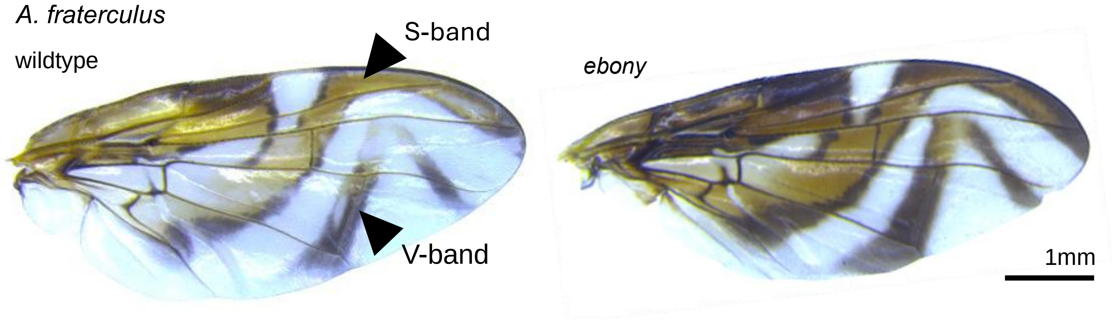

Supplementary Figure 4. (a) SANGER sequencing genotyping of *ebony* mutants in *A. fraterculus* G2. Ten out of 36 genotyped flies showed unambiguous results, all displaying a -7 bp deletion resulting in premature stop codons in exon 1 (frameshift mutation). A homozygous mutant line was subsequently established based on this same mutation. **(b)** Disruption of *ebony* leads to alterations in wing pigmentation of *A. fraterculus*, changing S- and V-bands from yellowish-brown to dark-brown with subtle pattern enlargement. No ectopic pigmentation was observed in the wing lamina. Terminology by White and Elson-Harris[1].

a

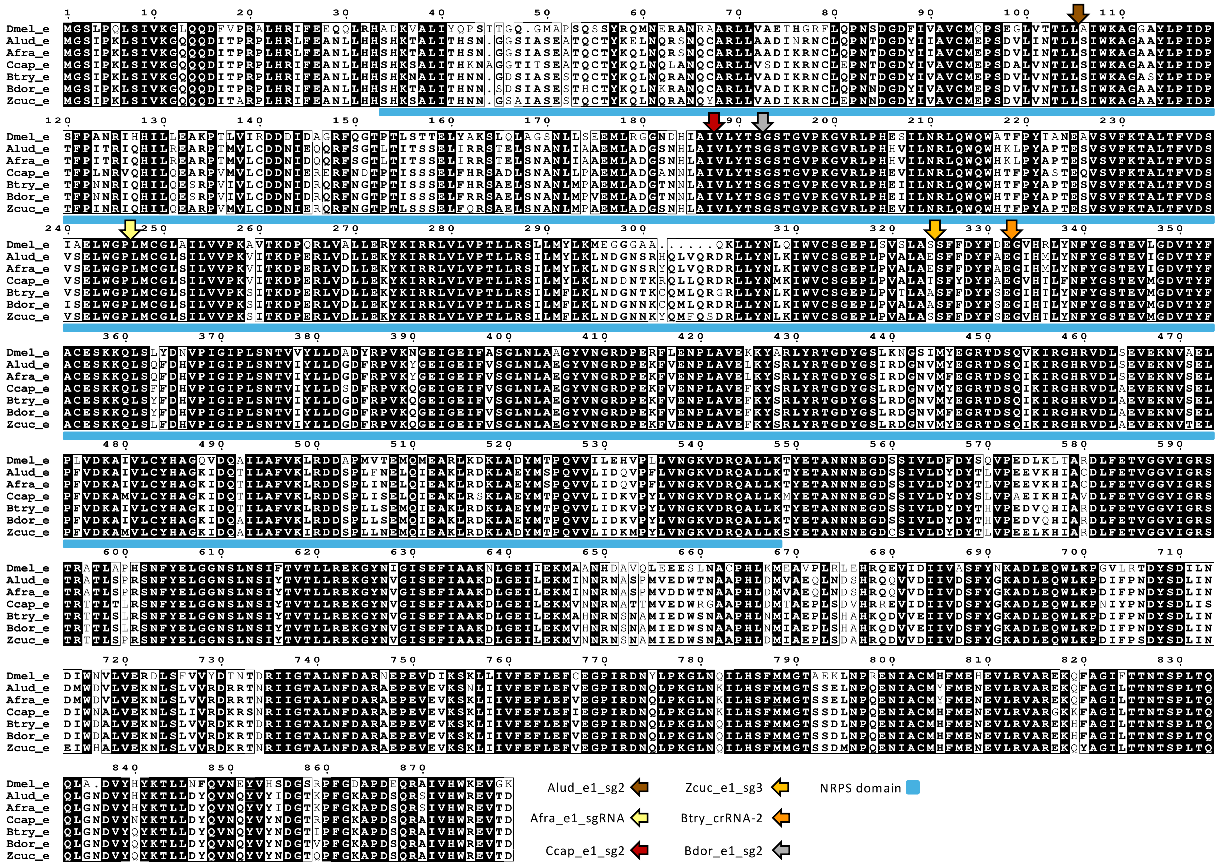

b

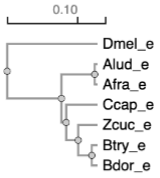

1: Dmel_e	100.00	72.55	72.67	72.35	73.69	73.69	73.80
2: Alud_e	72.55	100.00	99.10	88.37	89.50	89.16	89.50
3: Afra_e	72.67	99.10	100.00	88.49	89.73	89.62	89.62
4: Ccap_e	72.35	88.37	88.49	100.00	90.74	90.07	90.74
5: Btry_e	73.69	89.50	89.73	90.74	100.00	98.19	93.91
6: Bdor_e	73.69	89.16	89.62	90.07	98.19	100.00	94.02
7: Zcuc_e	73.80	89.50	89.62	90.74	93.91	94.02	100.00

Supplementary Figure 5. (a) Multiple sequence alignment of Ebony orthologues investigated in this study. Identical residues are shaded in black, while sites with equivalent residues (≥ 70% conservation) are in bold letters inside boxes. Arrowheads indicate approximate sgRNA targeted sites. The non-ribosomal peptide synthetase (NRPS) domain (S^{34} – K^{548}), responsible for the Ebony-catalyzed binding of dopamine to β-alanine[2,3], is underlined. **(b)** Phylogenetic relationship between Ebony orthologues (leftmost) and their genetic distance (rightmost). Manually curated gene models (Supplementary Data 1) were translated using the ExPASy, aligned with Clustal Omega, and visualized in ESPript. The Ebony sequence from *Drosophila* (FlyBase: FBgn0000527) was used as an outgroup. Key: Dmel = *Drosophila melanogaster*, Alud = *Anastrepha ludens*, Afra = *Anastrepha fraterculus*, Ccap = *Ceratitis capitata*, Zcuc = *Zeugodacus cucurbitae*, Btry = *Bactrocera tryoni*, and Bdor = *Bactrocera dorsalis*.

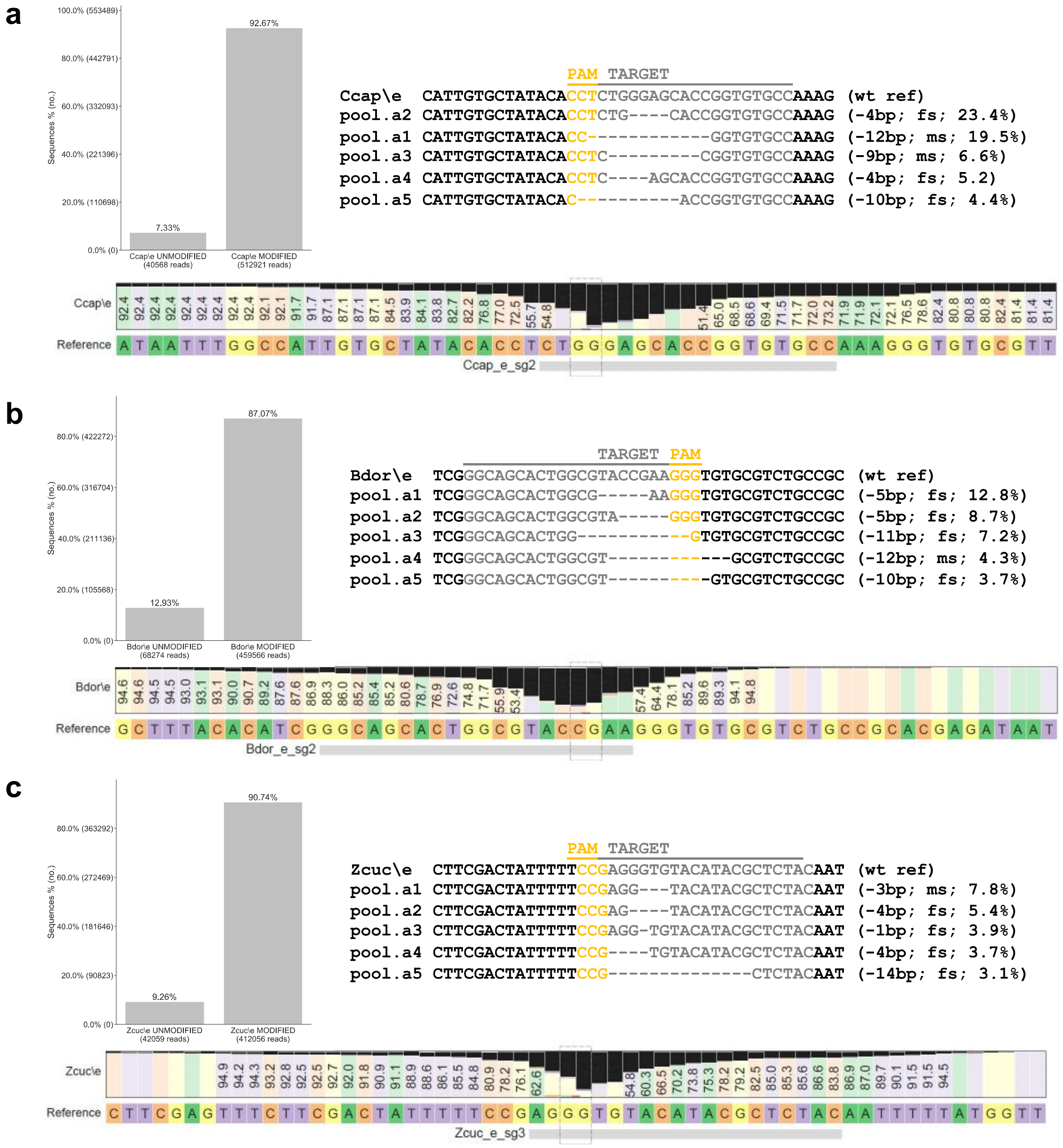

Supplementary Figure 6. Illumina genotyping of indexed amplicons surrounding Cas9 cut sites in G0 mosaic knockouts (mKO) of **(a)** *Ceratitis capitata*, **(b)** *Bactrocera dorsalis*, and **(c)** *Zeugodacus cucurbitae*. The CRISPResso2 pipeline was used for analysis (Quantification window center = -3, Quantification window size = 2 bp). Mutagenesis rate (leftmost) is defined as the percentage of reads displaying modified alleles, as shown in the most frequent variants (rightmost). Nucleotide distribution across the amplicon (below) reveals deletions (black bars) and insertions (brown bars) at the targeted sites. Each sample is a pool of mosaic G0 adults (n = 3). Percentages are relative to 454,115 – 553,489 mapped reads. Expected outcomes include a number of frameshift (fs) and missense (ms) mutations.

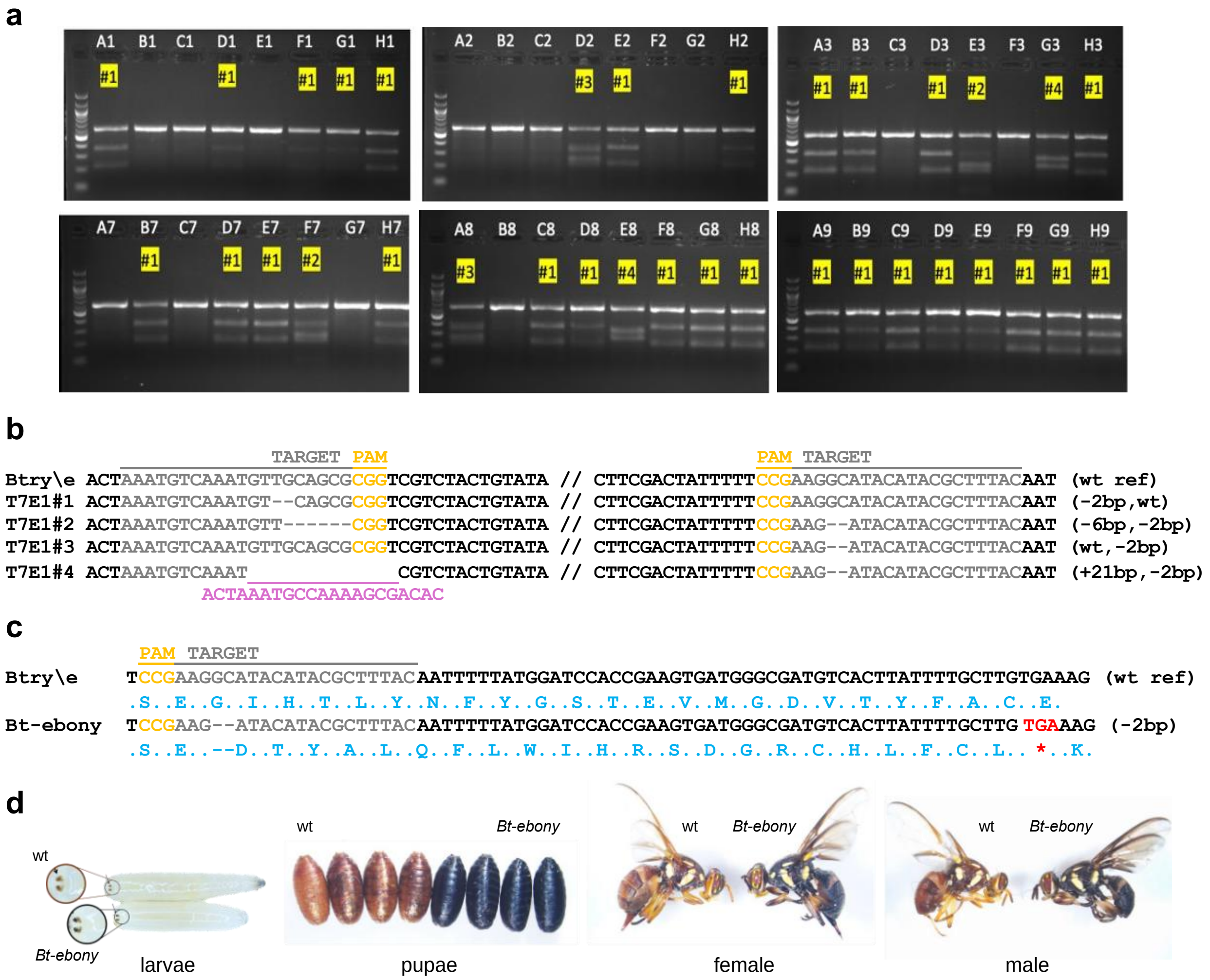

Supplementary Figure 7. Genotyping efforts during the establishment of a homozygous *ebony* mutant strain in *Bactrocera tryoni* (*Bt-ebony*). **(a)** T7 endonuclease I (T7EI) assays were used to identify heterozygous mutants from a pool of G2 individuals consisting of 24 males (A1 – H3) and 24 females (A7 – H9). Cleaved products indicate the presence of indels in the sample, and four distinct cleavage patterns were identified (highlighted in yellow). **(b)** SANGER sequencing of positive heterozygous G2 individuals revealed four *ebony* mutant alleles containing indels at one or both Cas9 targeted sites. **(c)** The homozygous mutant line *Bt-ebony* was established based on a -2 bp deletion, leading to premature stop codons in exon 1 (frameshift mutation) and thus *ebony* loss-of-function. **(d)** *ebony* mutants exhibit increased melanin production, resulting in black anal lobes in larvae, black puparium, and darker adult bodies compared to wildtype flies. As in other tephritids studied here, these phenotypes are comparable to the black pupae mutant in *A. ludens*[4].

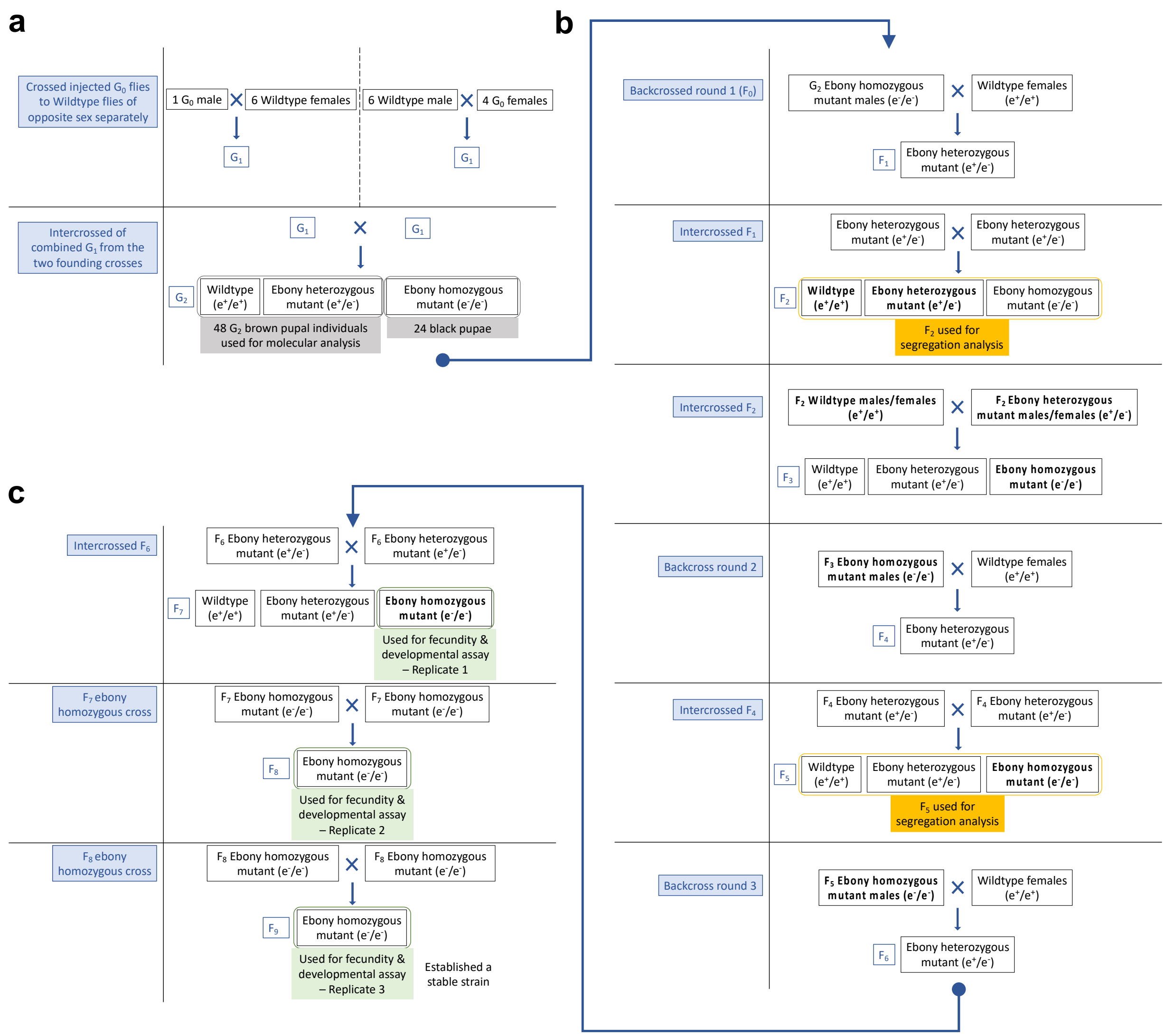

Supplementary Figure 8. Crossing scheme leading to a homozygous *ebony* mutant strain in *Bactrocera tryoni* (*Bt-ebony*). **(a)** Following microinjections, surviving G0 flies were backcrossed to their wildtype counterparts. All resulting G1 offspring were mass interbred, and *ebony* mutants recovered at G2. **(b)** To mitigate potential off-target effects from CRISPR/Cas9 experiments, homozygous *ebony* mutants harboring a -2 bp deletion were subjected to three additional rounds of backcrossing to the wildtype strain. Segregation analysis was conducted during the F2 and F5 generations. **(c)** Additional inbreeding was performed between homozygous mutants to establish the *Bt-ebony* strain. Fecundity and developmental assays were conducted with flies from the F7, F8, and F9 generations.

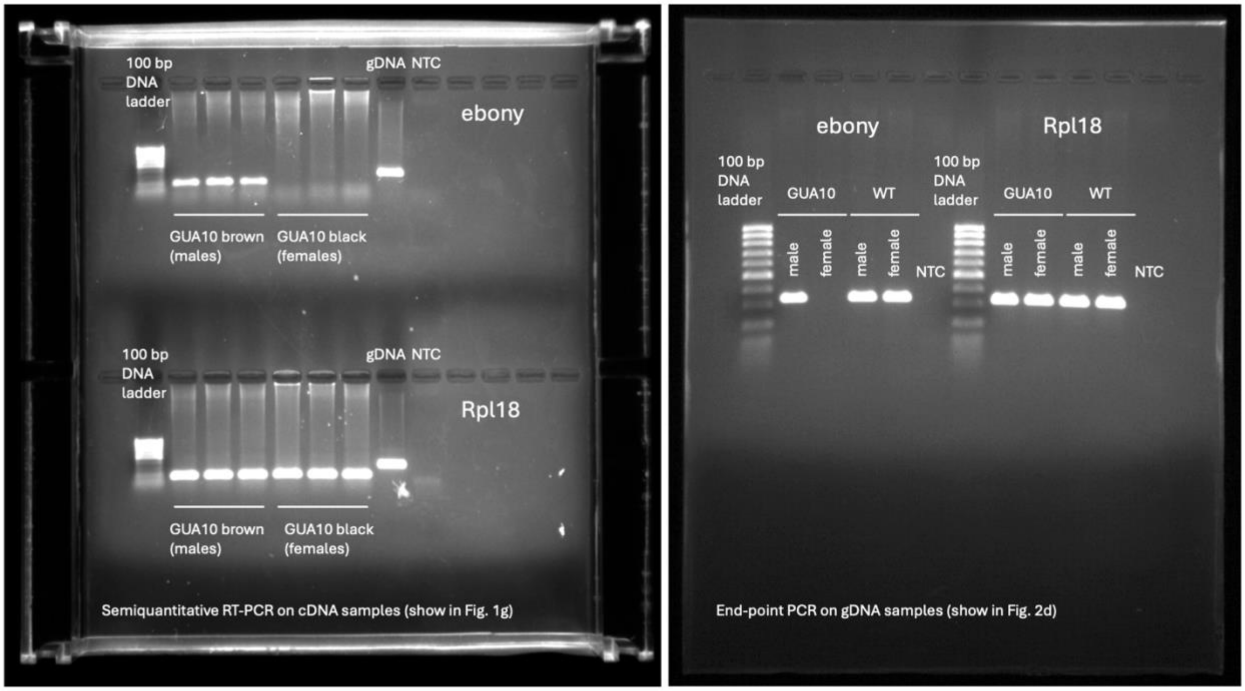

Supplementary Figure 9. Full-length images of cropped gels displayed in Fig. 1g and 2d. NTC = non-template control, gDNA = genomic DNA control, and WT = wildtype.

Supplementary Table 1. Gene Ontology (GO) terms for Biological Processes enriched in the bp causal region identified in the F4 mapping population. 'Annotated' indicates *A. ludens* genes annotated with the GO term. 'Significant' refers to genes in the causal region with the GO term. 'Expected' is the number of genes within the causal region with the GO term expected by sampling chance. Fisher exact test was used to calculate the *p*-values.

GO ID	Term	Annotated	Significant	Expected	*p*-value
GO:0007501	mesodermal cell fate specification	45	6	0.37	1.50E-06
GO:0021527	spinal cord association neuron differentiation	15	3	0.12	2.20E-04
GO:0051450	myoblast proliferation	15	3	0.12	2.20E-04
GO:0007483	genital disc morphogenesis	22	3	0.18	7.10E-04
GO:0007218	neuropeptide signaling pathway	88	5	0.72	7.20E-04
GO:0048665	neuron fate specification	51	4	0.41	7.60E-04
GO:0008340	determination of adult lifespan	323	9	2.63	1.17E-03
GO:0001709	cell fate determination	210	7	1.71	1.54E-03
GO:0010259	multicellular organism aging	343	9	2.79	1.78E-03
GO:0021522	spinal cord motor neuron differentiation	31	3	0.25	1.97E-03
GO:0030218	erythrocyte differentiation	69	4	0.56	2.34E-03
GO:0034243	regulation of transcription elongation by RNA polymerase II	35	3	0.28	2.81E-03
GO:0090110	COPII-coated vesicle cargo loading	10	2	0.08	2.82E-03
GO:0061320	pericardial nephrocyte differentiation	10	2	0.08	2.82E-03
GO:0017148	negative regulation of translation	178	6	1.45	3.19E-03
**GO:0006583**	**melanin biosynthetic process from tyrosine**	**11**	**2**	**0.09**	**3.42E-03**
GO:0048047	mating behavior, sex discrimination	11	2	0.09	3.42E-03
GO:0009408	response to heat	183	6	1.49	3.65E-03
GO:0042684	cardioblast cell fate commitment	12	2	0.1	4.09E-03
GO:0007368	determination of left/right symmetry	133	5	1.08	4.46E-03
GO:0042659	regulation of cell fate specification	83	4	0.67	4.57E-03
GO:0045893	positive regulation of DNA-templated transcription	896	15	7.29	5.11E-03
GO:0019184	nonribosomal peptide biosynthetic process	14	2	0.11	5.57E-03
GO:0007480	imaginal disc-derived leg morphogenesis	90	4	0.73	6.09E-03
GO:0071236	cellular response to antibiotic	145	5	1.18	6.42E-03
GO:0031327	negative regulation of cellular biosynthetic process	1172	21	9.53	7.21E-03
GO:0010628	positive regulation of gene expression	1321	19	10.74	8.20E-03
GO:0061418	obsolete regulation of transcription from RNA polymerase II promoter in response to hypoxia	17	2	0.14	8.20E-03
GO:1904385	cellular response to angiotensin	17	2	0.14	8.20E-03
GO:0045451	pole plasm oskar mRNA localization	53	3	0.43	9.05E-03
GO:0002474	antigen processing and presentation of peptide antigen via MHC class I	18	2	0.15	9.18E-03
GO:1904037	positive regulation of epithelial cell apoptotic process	18	2	0.15	9.18E-03
GO:0035050	embryonic heart tube development	102	4	0.83	9.41E-03
GO:0009064	glutamine family amino acid metabolic process	54	3	0.44	9.52E-03
GO:0051241	negative regulation of multicellular organismal process	781	13	6.35	9.71E-03

Supplementary Table 2. Differentially expressed genes (DEGs) between 1d-old black (*n* = 3, females) and brown (*n* = 3, males) pupae siblings of *A. ludens* GUA10 strain identified by edgeR. Overall, expression differences among 10,913 genes were not significant, with only four genes found to be upregulated in brown pupae samples. logFC = log2 fold-change between tested conditions (-*1*black 1*brown*), FDR = false discovery rate.

Feature	logFC	*p*-value	FDR	Gene	Chr. (RefSeq)	Function (FlyBase)
g794	8.2142	3.74E-07	0.0041	*Arc1*	6 (NC_071502.1)	Master regulator of synaptic plasticity (FBgn0033926).
g15354	7.4408	8.29E-06	0.0302	*ebony*	2 (NC_071498.1)	Links beta-alanine to biogenic amines like dopamine. Involved in cuticle pigmentation (FBgn0000527)
g13426	4.7262	1.16E-05	0.0316	unknown	2 (NC_071498.1)	Unknown. Also found in *Ceratitis* and *Bactrocera* (BLASTp).
g15461	1.6355	8.10E-06	0.0302	*Spc25*	2 (NC_071498.1)	Component of the Ndc80 complex, which is an essential kinetochore constituent (FBgn0087021)

Supplementary Table 3. Summary statistics of CRISPR/Cas9 experiments leading to the disruption of *ebony* in diverse tephritids. mKO = mosaic knockout, wt = wild-type.

Species	Eggs	Surviving G0 pupae			Crossing	Offspring (G1 or G2)		
		Total	Black	Brown		Total	Black	Brown
A. ludens	260	38 (14.6%)	4 (10.5%)	34 (89.5%)	mass G0 x G0	no data	none	all (100%)
A. fraterculus	179	24 (13.4%)	none	all (100%)	ind. G0 x wt → mass G1 x G1	no data	140	no data
C. capitata	260	69 (26.5%)	40 (58.0%)	29 (42.0%)	mass mKO x mKO	247	194 (78.5%)	53 (21.5%)
B. triony	661	12 (1.8%)	none	all (100%)	group G0 x wt → mass G1 x G1	no data	24	no data
B. dorsalis	240	82 (34.2%)	56 (68.3%)	26 (31.7%)	mass mKO x mKO	266	244 (91.7%)	22 (8.3%)
Z. cucurbitae	252	38 (15.1%)	26 (68.4%)	12 (31.6%)	mass mKO x mKO	441	266 (60.3%)	175 (39.7%)

Supplementary Table 4. Phenotype and genotype segregation analyses of *ebony* in *B. tryoni*. **(a)** F2 progeny from crosses between *B. tryoni ebony* mutants and wildtype flies exhibit no deviation from the expected 3:1 phenotype and 1:2 genotype ratios. Gender distribution based on F2 phenotypes **(b)** and wildtype brown pupae genotypes **(c)** in replicate 2 shows no bias in the expected 1:1 sex ratio.

a

**F0 parents**		**Rep.**	**F2 phenotypes**		**Chi-square against 3:1 ratio**			**F2 brown genotypes**		**Chi-square against 1:2 ratio**		
**Male**	**Female**		**brown**	**black**	**X2**	**df**	***p***	**e +/+**	**e +/-**	**X2**	**df**	***p*-value**
ebony	wildtype	#1	43	18	0.661	1	0.416	10	22	0.062	1	0.802
ebony	wildtype	#2	157	42	1.609	1	0.204	15	33	0.093	1	0.759
Overall stats			**200**	**60**	**0.513**	**1**	**0.474**	**25**	**55**	**0.156**	**1**	**0.692**

b

**F2 phenotypes**	**Sex**			**Chi-square against 1:1 ratio**		
**Rep. #2**	**Male**	**Female**	**Total**	**X2**	**df**	***p*-value**
brown	78	79	157	0.006	1	0.936
black	19	23	42	0.380	1	0.537
Overall stats	**97**	**102**	**199**	**0.125**	**1**	**0.723**

c

**F2 brown genotypes**	**Sex**			**Chi-square against 1:1 ratio**		
**Rep. #2**	**Male**	**Female**	**Total**	**X2**	**df**	***p*-value**
ebony +/+	8	7	15	0.666	1	0.796
ebony +/-	16	17	33	0.030	1	0.861
Overall stats	**24**	**24**	**48**	**0.000**	**1**	**1.000**

Supplementary Table 5. List of sequencing data from *A. ludens* used in this study. Dataset included sequences generated by Gutiérrez-Ramos et al[5]., Sirot et al.[6], and Congrains et al.[7]

SRA accession	Data	Sample	Purpose	Reference
SRR11028485	RNA-Seq	wildtype embryos 0-9h AEL	structural genome annotation	Gutiérrez-Ramos et al.
SRR11028488	RNA-Seq	wildtype embryos 9-18h AEL	structural genome annotation	Gutiérrez-Ramos et al.
SRR11028491	RNA-Seq	wildtype embryos 18-30h AEL	structural genome annotation	Gutiérrez-Ramos et al.
SRR8612579	RNA-Seq	GUA10 female pupa	structural genome annotation	*unpublished data*
SRR8612578	RNA-Seq	GUA10 male pupa	structural genome annotation	*unpublished data*
SRR8612576	RNA-Seq	wildtype virgin female	structural genome annotation	*unpublished data*
SRR8612577	RNA-Seq	wildtype mated female	structural genome annotation	*unpublished data*
SRR9841864	RNA-Seq	wildtype naive male	structural genome annotation	Sirot et al.
SRR9841863	RNA-Seq	wildtype mated male	structural genome annotation	Sirot et al.
SRR29931067, SRR29931113 - SRR29931123, SRR29931129, SRR29931130, SRR29931132, SRR29931137, SRR29931142, SRR29931143	WGS	F4 mapping population, brown pupae	identification of the bp causal region	this study
SRR29931069 - SRR29931076, SRR29931078 - SRR29931087	WGS	F4 mapping population, black pupae	identification of the bp causal region	this study
SRR30148297 – SRR30148299	RNA-Seq	GUA10 black pupae female	DGE analysis	this study
SRR30148294 – SRR30148296	RNA-Seq	GUA10 brown pupae male	DGE analysis	this study
SRR17880705	HiFi-Seq	wildtype (Willacy) adult male	characterization of the *bp* mutation	Congrains et al.
SRR30151613	HiFi-Seq	GUA10 adult female	characterization of the *bp* mutation	this study
SRR30151612	HiFi-Seq	GUA10 brown adult male	characterization of the *bp* mutation	this study

Supplementary Table 6. List of primers used in this study.

Primer name	Sequence (5' → 3')	Purpose
Alud_e_F1	GCCAGTTCGATCATGTACCG	PCR (360bp) and RT-PCR (188bp) spanning Alud\e exons e1 and e2
Alud_e_R2	TTCCACAGCCAATGGATTTTCG	
Alud_RpL18_F1	AACTGAGCCCAAATCGCAAG	PCR (351bp) and RT-PCR (156bp) spanning Alud\RpL18 exons e1 and e2
Alud_RpL18_R1	CTGACACGCTGCAAAGACAT	
Alud_ebony_probe_new_F	CTGGCTCAATTGCTTTTTGCT	PCR generated *in situ* probe for Alud\e
Alud_ebony_probe_new_R	AACTGCACTGATAACGCACAG	
Alud_e1_sg2	GAAATTAATACGACTCACTATAGGGGTCA ATTGGTAGATAGGGTTTTAGAGCTAGAAA TAGC	synthesis of sgRNA against Alud\e
Alud_e1_sg2_fwd.p5	TCGTCGGCAGCGTCAGATGTGTATAAGA GACAGTGTGCTCATCAATACGCTGC	Illumina genotyping of PCR amplicons (173bp) spanning guide recognition site in Alud\e
Alud_e1_sg2_rev.p7	GTCTCGTGGGCTCGGAGATGTGTATAAG AGACAGTAATGGTGAGTGTGCCGCTA	
Afra_e1_sgRNA	ATAGAAAGTCCACACATCAG	custom sgRNA against Afra\e
GMB_174_rev	ACATCGCCTATCACTTCGGT	SANGER genotyping of PCR amplicons spanning guide recognition site in Afra\e
GMB_179_fwd	GCACAACAATGGCGGTTCAA	
Ccap_e1_sg2	GAAATTAATACGACTCACTATAGGCACAC CGGTGCTCCCAGGTTTTAGAGCTAGAAA TAGC	synthesis of sgRNA against Ccap\e.
Ccap_e1_sg2_fwd.p5	TCGTCGGCAGCGTCAGATGTGTATAAGA GACAGCAGCAGCTCGGAACTCTTTC	Illumina genotyping of PCR amplicons (211bp) spanning guide recognition site in Ccap\e
Ccap_e1_sg2_rev.p7	GTCTCGTGGGCTCGGAGATGTGTATAAG AGACAGCTCTGTGGACGCATAAGGGA	
Btry_crRNA-1	AAATGTCAAATGTTGCAGCG	custom crRNA against Btry\e
Btry_crRNA-2	GTAAAGCGTATGTATGCCTT	custom crRNA against Btry\e
Qebony_F1	CAAGACCGCACTCACCTTTG	T7E1 and SANGER genotyping of PCR amplicons spanning guide recognition sites in Btry\e
Qebony_R1	CTCCTTGTTTGACTGGTCGG	
Bdor_e1_sg2	GAAATTAATACGACTCACTATAGGCAGCA CTGGCGTACCGAAGTTTTAGAGCTAGAA ATAGC	synthesis of sgRNA against Bdor\e
Bdor_e1_sg2_fwd.p5	TCGTCGGCAGCGTCAGATGTGTATAAGA GACAGACGCTAATTTGATGCCGGYC	Illumina genotyping of PCR amplicons (198bp) spanning guide recognition site in Bdor\e
Bdor_e1_sg2_rev.p7	GTCTCGTGGGCTCGGAGATGTGTATAAG AGACAGTGAGTGCGGTCTTGAAAACG	
Zcuc_e1_sg3	GAAATTAATACGACTCACTATAGGTAGAG CGTATGTACACCCTGTTTTAGAGCTAGAA ATAGC	synthesis of sgRNA against Zcuc\e
Zcuc_e1_sg3_fwd.p5	TCGTCGGCAGCGTCAGATGTGTATAAGA GACAGCCGTTTAGTTTTGGTGCCGA	Illumina genotyping of PCR amplicons (237bp) spanning guide recognition site in Zcuc\e
Zcuc_e1_sg3_rev.p7	GTCTCGTGGGCTCGGAGATGTGTATAAG AGACAGACGTCACCCATTACTTCRGT	

Supplementary References

1. White, I. M. & Elson-Harris, M. M. *Fruit Flies of Economic Significance: Their Identification and Bionomics.* (CAB international, 1992).
2. Richardt, A. *et al.* Ebony, a novel nonribosomal peptide synthetase for β-alanine conjugation with biogenic amines in Drosophila. *Journal of Biological Chemistry* **278**, 41160–41166 (2003).
3. Platt, A. J., Padrick, S., Ma, A. T. & Beld, J. A dissected non-ribosomal peptide synthetase maintains activity. *Biochimica et Biophysica Acta (BBA)-Proteins and Proteomics* **1872**, 140972 (2024).
4. Zepeda-Cisneros, C. S. *et al.* Development, genetic and cytogenetic analyses of genetic sexing strains of the Mexican fruit fly, Anastrepha ludens Loew (Diptera: Tephritidae). *BMC Genomic Data* **15**, 1–11 (2014).
5. Gutiérrez-Ramos, X. *et al.* Novel tephritid-specific features revealed from cytological and transcriptomic analysis of Anastrepha ludens embryonic development. *Insect Biochemistry and Molecular Biology* **122**, 103412 (2020).
6. Sirot, L. *et al.* Post-mating gene expression of Mexican fruit fly females: Disentangling the effects of the male accessory glands. *Insect Molecular Biology* **30**, 480–496 (2021).
7. Congrains, C. *et al.* Chromosome-scale genome of the polyphagous pest Anastrepha ludens (Diptera: Tephritidae) provides insights on sex chromosome evolution in Anastrepha. *G3 Genes|Genomes|Genetics* jkae239 (2024) doi:10.1093/g3journal/jkae239.
