# Supplementary material for: Functional genomics implicates ebony in the black pupae phenotype of tephritid fruit flies
Source: Commun Biol. 2025 Jan 15;8:60. doi: 10.1038/s42003-025-07489-y (PMC11736145; doi:10.1038/s42003-025-07489-y)
Supplement: Supplementary file 2 — Description of Additional Supplementary Materials [file 42003_2025_7489_MOESM2_ESM.pdf]

# Description of Additional Supplementary Files

**File name:** Supplementary Data 1

**Description:** Manually curated annotation of ebony orthologues investigated in this study.

**File name:** Supplementary Data 2

**Description:** TMM-normalized RPKM read counts.

**File name:** Supplementary Data 3

**Description:** Source data for fitness analysis of Btebony mutants.
