# Supplementary material for: Functional genomics implicates ebony in the black pupae phenotype of tephritid fruit flies
Source: Commun Biol. 2025 Jan 15;8:60. doi: 10.1038/s42003-025-07489-y (PMC11736145; doi:10.1038/s42003-025-07489-y)
Supplement: Supplementary file 6 — Reporting Summary [file 42003_2025_7489_MOESM6_ESM.pdf]

nature portfolio

Corresponding author(s): Scott M. Geib, PhD (scott.geib@usda.gov)

Last updated by author(s): Dec 13, 2024

Reporting Summary

Nature Portfolio wishes to improve the reproducibility of the work that we publish. This form provides structure for consistency and transparency in reporting. For further information on Nature Portfolio policies, see our Editorial Policies and the Editorial Policy Checklist.

Statistics

For all statistical analyses, confirm that the following items are present in the figure legend, table legend, main text, or Methods section.

n/a	Confirmed	
☐	☒	The exact sample size (*n*) for each experimental group/condition, given as a discrete number and unit of measurement
☐	☒	A statement on whether measurements were taken from distinct samples or whether the same sample was measured repeatedly
☐	☒	The statistical test(s) used AND whether they are one- or two-sided<br>*Only common tests should be described solely by name; describe more complex techniques in the Methods section.*
☐	☒	A description of all covariates tested
☐	☒	A description of any assumptions or corrections, such as tests of normality and adjustment for multiple comparisons
☐	☒	A full description of the statistical parameters including central tendency (e.g. means) or other basic estimates (e.g. regression coefficient) AND variation (e.g. standard deviation) or associated estimates of uncertainty (e.g. confidence intervals)
☐	☒	For null hypothesis testing, the test statistic (e.g. *F*, *t*, *r*) with confidence intervals, effect sizes, degrees of freedom and *P* value noted<br>*Give P values as exact values whenever suitable.*
☒	☐	For Bayesian analysis, information on the choice of priors and Markov chain Monte Carlo settings
☒	☐	For hierarchical and complex designs, identification of the appropriate level for tests and full reporting of outcomes
☒	☐	Estimates of effect sizes (e.g. Cohen's *d*, Pearson's *r*), indicating how they were calculated

Our web collection on statistics for biologists contains articles on many of the points above.

Software and code

Policy information about availability of computer code

Data collection

no software was used for data collection

Data analysis

The following is a list of all relevant software used in this study. All parameters (other than default) and further details can be found in the Materials and Methods section of the manuscript.

(1) Genomics:
fgbio toolkit: Demultiplex raw reads
fastp v0.23.2: Raw data clean-up
BWA-MEM v.2.2.1: WGS mapping (alignment)
SAMtools v1.17 and SAMBLASTER v0.1.26: WGS alignment manipulation
GATK v.4.4 and VCFtools v0.1.16-9: Variant calling
R v4.3.2 (package qqman) and IGV v2.16.2-0: Visualization
RepeatModeler v2.0.4 and RepeatMasker v4.1.5: Mask reference genome
STAR v2.7.10b: RNA-Seq mapping (alignment)
BRAKER2 and AGAT v0.8.0: Genome feature annotations
BUSCO v5.4.5: Genome annotation evaluation
BLASTp, InterProScan v5.64-96.0, and eggNOG-mapper v2.1.12-0: Genome functional annotations
topGO v2.54.0-0: GO enrichment analysis
SMRTLink v13.0: Get HiFi circular consensus
HiFiAdapterFilt v.3.0.1: Filter raw HiFi data
minimap2 v.2.24: Mapping HiFi data to reference genome

tBLASTn, BEDTools v2.31.1-0, and Exonerate v2.4.0-7: Manual curation of gene annotations
CRISPOR v.5.01 and CHOPCHOP v.3: Design of sgRNAs
CRISPResso2 v2.2.14-0: Genotyping of CRISPR G0 flies
Geneious v.2023.0.2: General sequence analysis

(2) Transcriptomics:
fastp v0.23.2: Raw data clean-up
STAR v2.7.10b: RNA-Seq mapping (alignment)
featureCounts (subread v2.0.4): RNA-Seq count matrix (raw expression data)
edgeR v4.0.12: DEG analysis

(3) Others:
Image Lab v.6.0.1: Gel analysis
LAS X software v3.7.0: Cytogenetics analysis
R v.4.3.2 (package stats): Statistical analysis

For manuscripts utilizing custom algorithms or software that are central to the research but not yet described in published literature, software must be made available to editors and reviewers. We strongly encourage code deposition in a community repository (e.g. GitHub). See the Nature Portfolio guidelines for submitting code & software for further information.

Data

Policy information about availability of data

All manuscripts must include a data availability statement. This statement should provide the following information, where applicable:

- Accession codes, unique identifiers, or web links for publicly available datasets
- A description of any restrictions on data availability
- For clinical datasets or third party data, please ensure that the statement adheres to our policy

The following reference genome assemblies were used in this study: A. ludens (GenBank: GCA_028408465.1), C. capitata (GenBank: GCA_000347755.4), B. dorsalis (GenBank: GCA_023373825.1), B. tryoni (GenBank: GCA_016617805.2), and Z. cucurbitae (GenBank: GCA_028554725.2). All raw sequencing data generated in this study are deposited in the NCBI Sequence Read Archive (SRA) database under the BioProject PRJNA1139181. SRA accession numbers for each sample are detailed in Supplementary Table 5. Final de novo genome annotation files for A. ludens are archived on figshare repository at https://doi.org/10.6084/m9.figshare.26376841. All primers used in this study are listed in Supplementary Table 6. Manually curated annotations of ebony orthologues can be found in Supplementary Data 1. TMM-normalized RPKM expression data can be found in Supplementary Data 2. The source data for fitness analysis of B. tryoni ebony strain is available as Supplementary Data 3. Full-length gels are shown in Supplementary Fig. 9.

Research involving human participants, their data, or biological material

Policy information about studies with human participants or human data. See also policy information about sex, gender (identity/presentation), and sexual orientation and race, ethnicity and racism.

Reporting on sex and gender	Not applicable
Reporting on race, ethnicity, or other socially relevant groupings	Not applicable
Population characteristics	Not applicable
Recruitment	Not applicable
Ethics oversight	Not applicable

Note that full information on the approval of the study protocol must also be provided in the manuscript.

Field-specific reporting

Please select the one below that is the best fit for your research. If you are not sure, read the appropriate sections before making your selection.

☒ Life sciences ☐ Behavioural & social sciences ☐ Ecological, evolutionary & environmental sciences

For a reference copy of the document with all sections, see nature.com/documents/nr-reporting-summary-flat.pdf

Life sciences study design

All studies must disclose on these points even when the disclosure is negative.

Sample size	For WGS mapping, the sample size was determined by the number of F4 offspring (from a single family; isofemale crossing) exhibiting the experimental phenotypes (brown or black pupae), with sampling balanced based on the number of recessive mutants obtained (see "Data exclusions" for more details). For phenotypic analysis of C. capitata, B. dorsalis, and Z. cucurbitae, as well as fitness analysis of B. tryoni, all

	offspring from genetic crossings were used.
Data exclusions	In total, we sequenced 78 WGS libraries from the F4 mapping population, including 60 brown pupae adults (76.9%) and 18 black pupae adults (23.1%), roughly as expected by the 3:1 Mendelian inheritance ratio of phenotypes (X2 =0.054, p =0.815, d.f=1). However, to have a more balanced ratio of phenotypes for the FST analysis, we selected only 18 samples from the brown pupae group, using the sequencing data with the most raw reads. This exclusion was pre-established before WGS read mapping.
Replication	RNA-Seq was performed in triplicate, which we considered the minimum acceptable number for robust statistical analysis in edgeR. Semiquantitative end-point PCRs were conducted on the same samples used for transcriptomics (n = 3 for each group) and were run in technical triplicates. Negative and positive amplification controls were also included whenever possible. The large deletion at the ebony locus, initially found in the F4 mapping population, was later confirmed in flies from the original GUA10 strain currently reared at MOSCAMED in Guatemala. For comparative phenotypic analysis, a large number of individuals (n > 50) per species were analyzed. Multiple Cas9-mediated KO individuals or pools of flies were used for genotyping by sequencing and/or T7E1 assays. Fitness analysis, including segregation and fecundity tests, was conducted over three subsequent generations to account for environmental variations. Different combinations of crossings (n = 4) using B. tryoni ebony mutants and wild-type flies were also performed in triplicate to address these variations.
Randomization	Samples were allocated into experimental groups based on their pupae phenotype (brown or black) for all experiments carried out in this study. Sex and genotypes were covariates in fitness experiments with B. tryoni.
Blinding	Blinding was irrelevant for most of this study, as we investigated the differences between two well-defined groups based on visible phenotypes. However, we were blinded regarding brown pupae genotypes during WGS mapping and fitness analysis.

Reporting for specific materials, systems and methods

We require information from authors about some types of materials, experimental systems and methods used in many studies. Here, indicate whether each material, system or method listed is relevant to your study. If you are not sure if a list item applies to your research, read the appropriate section before selecting a response.

Materials & experimental systems

n/a	Involved in the study
☒	☐ Antibodies
☒	☐ Eukaryotic cell lines
☒	☐ Palaeontology and archaeology
☐	☒ Animals and other organisms
☒	☐ Clinical data
☒	☐ Dual use research of concern
☒	☐ Plants

Methods

n/a	Involved in the study
☒	☐ ChIP-seq
☒	☐ Flow cytometry
☒	☐ MRI-based neuroimaging

Animals and other research organisms

Policy information about studies involving animals; ARRIVE guidelines recommended for reporting animal research, and Sex and Gender in Research

Laboratory animals	The following strains of tephritids were used in this study: Anastrepha ludens (GUA10 and WT rearing strains), Anastrepha fraterculus sp. 1 (WT Vacaria strain), Ceratitis capitata (WT rearing strain), Bactrocera dorsalis (WT Punador strain), Bactrocera tryoni (WT Ourimbah strain), and Zeugodacus cucurbitae (WT rearing strain). These species were analyzed at various developmental stages (from eggs to adults), as described in the Materials and Methods section of the manuscript. Details of each sample used in WGS and RNA-Seq mappings can also be found in NCBI BioSamples under the BioProject PRJNA1139181. Briefly, microinjections were conducted in eggs, pupae were used for RNA-Seq (GUA10), and adults were used for genetic crossings, FST analysis, sequencing, and phenotype analysis.
Wild animals	This study did not involved wild animals collected in the field.
Reporting on sex	The main finding of this study was linking the ebony gene to the black pupae phenotype in tephritids. The black pupae mutation is a known autosomal recessive marker; therefore, no sex-based analysis was required. However, results from genetic crossings of B. tryoni can be found in Supplementary Table 4, where we compared the number of male and female offspring against the expected 1:1 sex ratio. Bactrocera species–as well as most tephritid flies–can easily be sorted by sex due to the presence of female ovipositors. B. tryoni Males and females were also sorted prior to crossings made for fitness analysis using the same method.
Field-collected samples	This study did not involved samples collected from the field.
Ethics oversight	This work was developed using tephritid fruit flies as model organisms, which—similarly to Drosophila—does not require ethical approval according to the guidelines provided by our institutions. However, best rearing and sampling practices were followed to minimize harm and distress and to ensure proper care.

Note that full information on the approval of the study protocol must also be provided in the manuscript.

Plants

Seed stocks	Not applicable
Novel plant genotypes	Not applicable
Authentication	Not applicable
